# Supplementary material for: Behavioral and electrophysiological responses of Hippodamia variegata to plant volatiles
Source: PLoS One. 2025 Jun 30;20(6):e0327160. doi: 10.1371/journal.pone.0327160 (PMC12208412; doi:10.1371/journal.pone.0327160)
Supplement: Attachment — Submitted filename: response.docx [file pone.0327160.s002.docx]

Dear Editor,

Thank you for your guidance regarding the Funding Statement. As requested, I have included the amended funding declaration within the cover letter below. Please let me know if any additional information is needed.

This work was supported by the National Key R&D Program of China (2022YFD1400300), and China Agriculture Research System of MOF and MARA (CARS-15-19).

I appreciate your assistance in updating the online submission form on my behalf. Kindly confirm if further action is required from my side.

Thank you for your support throughout the submission process.

Sincerely,

Yanhui Lu

Dear Editors and Reviewers:

Thanks for your letter and constructive comments concerning our manuscript entitled “Behavioral and electrophysiological responses of *Hippodamia variegata* to plant volatiles” (ID: PONE-D-25-17465). We greatly appreciate your complimentary comments and suggestions. Based on your suggestions, we have made corrected modifications throughout the manuscript.

We hope the present version meet the Journal’s approval. The point-to-point responses were listed below.

Sincerely,

Yanhui Lu

**Journal requirements:**

1. Please ensure that your manuscript meets PLOS ONE's style requirements, including those for file naming. The PLOS ONE style templates can be found at

https://journals.plos.org/plosone/s/file?id=wjVg/PLOSOne_formatting_sample_main_body.pdf and

[https://journals.plos.org/plosone/s/file?id=ba62/PLOSOne_formatting_sample_title_authors_affiliations.pdf](https://track.editorialmanager.com/CL0/https:%2F%2Fjournals.plos.org%2Fplosone%2Fs%2Ffile%3Fid=ba62%2FPLOSOne_formatting_sample_title_authors_affiliations.pdf/1/010f0196f4318b5e-36ed479e-e23c-4892-8c33-2c14bf084d89-000000/vSxSQ5dOMIQlUbXEHn96cZPynTrnY8u3A0r2pFQdnQk=212)

**Reply:** We have made modifications according to PLOS ONE's style.

2. Thank you for stating in your Funding Statement:

 [This work was supported by the National Key R&D Program of China (2022YFD1400300), and China Agriculture Research System of MOF and MARA (CARS-15-19).].

Please provide an amended statement that declares *all* the funding or sources of support (whether external or internal to your organization) received during this study, as detailed online in our guide for authors at http://journals.plos.org/plosone/s/submit-now.  Please also include the statement “There was no additional external funding received for this study.” in your updated Funding Statement.

Please include your amended Funding Statement within your cover letter. We will change the online submission form on your behalf.

**Reply:** Thank you for your guidance regarding the Funding Statement. As requested, I have included the amended funding declaration within the cover letter below. Please let me know if any additional information is needed.

3. In the online submission form, you indicated that [Data will be made available on request.].

All PLOS journals now require all data underlying the findings described in their manuscript to be freely available to other researchers, either 1. In a public repository, 2. Within the manuscript itself, or 3. Uploaded as supplementary information.

This policy applies to all data except where public deposition would breach compliance with the protocol approved by your research ethics board. If your data cannot be made publicly available for ethical or legal reasons (e.g., public availability would compromise patient privacy), please explain your reasons on resubmission and your exemption request will be escalated for approval.

**Reply:** We will provide access to the raw experimental data underlying this manuscript, with detailed datasets available in the supplementary files.

**Reviewer #1:**

1. The results are overall clearly presented.

**Reply:** Thank you for your positive feedback on the clarity of the results presentation. We are pleased to know that the organization and interpretation of the data met the journal's standards.

If there are any specific sections within the Results that require further elaboration or refinement to enhance readability, please do not hesitate to let us know. We are fully prepared to make additional revisions as needed.

2. A regression analysis quantifying the EAG response-concentration relationship for each compound would strengthen the statistical interpretation (e.g., linear vs. nonlinear trends).

**Reply:** Thanks. The relationship between substance concentration and the olfactory response of the lady beetle may not be linear. In our experiment, we tested six concentration gradients. Behavioral data revealed that the selectivity of *H. variegata* did not consistently increase with rising concentrations. Specifically, their olfactory response did not necessarily intensify at higher concentrations, and beyond a certain threshold, the response may stabilize. Therefore, we conclude that adding regression lines with R²/p-values to the graph would be inappropriate.

3. The role of methyl jasmonate and methyl dihydrojasmonate as volatile plant defense signals (e.g., induced emission in undamaged plants) and how this might explain discrepancies between EAG and behavioral assays should be mentioned in discussion. Relevant citations should be incorporated.

**Reply:** We sincerely appreciate the valuable comments. We have incorporated the pertinent discussions into the revised manuscript and supplemented corresponding references to support these additions. L268-271.

4. Original data used for statistics were not provided.

**Reply:** We will provide access to the raw experimental data underlying this manuscript, with detailed datasets available in the supplementary files.

Minor comments
Line 128 and 129: the dash in these words “de-scribed” and “con-centration” are not necessary.

**Reply:** Thank you for your valuable suggestions. We have made the corresponding revisions in the manuscript.

Line 142: “Beetle larvae were tested by also connecting one end of the antenna to the reference electrode and the other end to the recording electrode, as described above.” Clarify that larval EAG data were not included (to avoid confusion).

**Reply:** Thank you for your feedback. This study did not involve any larval experiments. We have revised the relevant content in the manuscript.

Line 158: “A. gossypii” should be in italic.

**Reply:** Thanks. A. gossypii has been formatted in italics.

Line 159-160: Specify filter paper height (e.g., "10 cm above the soil surface").

**Reply:** Thank you for your suggestion. We have revised the sentence to “Subsequently, 20 μL of the test compound (10 μg/μL) was dropwise applied onto filter paper positioned 10 cm above the soil surface of an individual plant.” L160-161.

Line 265-267: methyl jasmonate and methyl dihydrojasmonate are volatile signals that can induce defense responses in undamaged plants and enhance their volatile emission. This induction might also be one reason for the difference between EAG responses and the behavioral responses. The discussion should be improved and citations about this point should be included.

**Reply:** Thanks. We have added this content to the discussion section and included relevant references. The added content is as follows: Methyl jasmonate and methyl dihydrojasmonate act as volatile signaling compounds, triggering defense responses in intact plants and amplifying their volatile organic compound (VOC) emissions. This systemic induction may partially account for the observed discrepancy between EAG responses and behavioral responses. L268-271.

Figure 3. Add regression lines with R²/p-values to visualize volatile concentration-EAG response relationships.

**Reply:** The relationship between substance concentration and the olfactory response of the lady beetle may not be linear. In our experiment, we tested six concentration gradients. Behavioral data revealed that the selectivity of *H. variegata* did not consistently increase with rising concentrations. Specifically, their olfactory response did not necessarily intensify at higher concentrations, and beyond a certain threshold, the response may stabilize. Therefore, we conclude that adding regression lines with R²/p-values to the graph would be inappropriate.

How about the effect of volatile compound mixture? Future Work: testing compound mixtures could reveal synergistic effects (useful for field applications).

**Reply:** Thank you for your question. The effects of volatile mixtures are indeed a key focus of our future research. In subsequent experiments, we will prioritize observing their performance under field conditions, as examining compound mixtures could reveal potential synergistic effects. We have also expanded the discussion section to address this topic. L297-298.

Reviewer #2:

1. line 69, ‘Mentha’ need to be italic.

**Reply:** Thanks. We have italicized ‘Mentha’.

2. line 126, it is better to introduce why select 100, 10, and 1 μg/μL these three concentration

**Reply:** In chemical ecology research, using concentration gradients of 1, 10, and 100 μg/μL to conduct insect behavioral response tests is a widely adopted standard method. For example, Jiménez-Santiago et al. (2024) employed dose gradients of 1, 10, and 100 ng in their study on the behavioral responses of *Triatoma pallidipennis* to volatile substances, systematically evaluating the effects of different concentrations on insect attraction and repellence in a Y-tube olfactometer. We have added the corresponding references in the manuscript. L126.

3. line 158, 'A. gossypii' need to be italic.

**Reply:** Thanks. We have italicized 'A. gossypii'.

4. line 205-206, between 100 and μg/μL, one space is be lack. please check other place in text.

**Reply:** Thank you for highlighting the minor formatting issues. We have made the necessary corrections and reviewed the entire article for similar minor issues.

5. line 'By comparing their P-values, ... have better attraction effects on the male H. variegata at 10 μg/μL than at 100μg/μL.' The size of the number of P-values between two treatment to CK could to be used to deduce the difference of those two events?

**Reply:** The p-value is a statistical parameter used to determine the results of hypothesis testing. It represents the probability of observing the current result or more extreme outcomes under the assumption that the null hypothesis is true. A smaller p-value provides stronger justification to reject the null hypothesis and conclude that differences exist between the compared groups. In this experiment, the p-value partially reflects the level of ladybug selection rate; generally, a smaller p-value indicates higher selectivity in ladybug behavior. Therefore, the p-values between the two experimental treatments and the control group (CK) can be directly compared to evaluate differences.
